# Rationale, design, and methodology for the healthy mothers-healthy children study: a randomized controlled trial

**DOI:** 10.1186/s40795-019-0322-7

**Published:** 2019-12-16

**Authors:** Diane C. Berry, Cecilia Gonzales, Nilda Peragallo Montano, Krista M. Perreira, Alice S. Ammerman, Jaime Crandell, Kelly R. Evenson, Myles S. Faith, Hugh Waters, Crystal Linares, Yamnia I. Cόrtes, Eliana M. Perrin

**Affiliations:** 1 School of Nursing, The University of North Carolina at Chapel Hill, Campus Box 7460, Chapel Hill, North Carolina 27599-7460 USA; 2 Department of Social Medicine, The University of North Carolina at Chapel Hill, 333 South Columbia Street, MacNider Hall, Room #348 / CB #7240, Chapel Hill, NC 27599-7240 USA; 3 Gillings School of Global Public Health, Department of Nutrition, The University of North Carolina at Chapel Hill, CB#7426, Chapel Hill, NC 27599 USA; 4 Center for Health Promotion and Disease Prevention, The University of North Carolina at Chapel Hill, CB#7426, Chapel Hill, NC 27599 USA; 5 School of Nursing, The University of North Carolina at Chapel Hill, Campus Box 7460, Chapel Hill, North Carolina 27599-8050 USA; 6 Gillings School of Global Public Health, Department of Biostatistics, The University of North Carolina at Chapel Hill, Campus Box 7460, Chapel Hill, North Carolina 27599-8050 USA; 7 Gillings School of Global Public Health, Department of Epidemiology, The University of North Carolina at Chapel Hill, Campus Box 8050, Chapel Hill, North Carolina 27599-8050 USA; 8 School and Educational Psychology, University of Buffalo Graduate School of Education, 420 Baldy Hall, North Campus, Buffalo, New York 14260-1000 USA; 9 Health Economics Research, Milken Institute, 1250 Fourth Street, Santa Monica, California 90401 USA; 10 Division of Primary Care, Department of Pediatrics, Duke Center for Childhood Obesity Research, Duke University School of Medicine, DUMC 102645, 146 Civitan Building, 2213 Elba Street, Durham, NC 27705 USA

**Keywords:** Mothers, Children, Nutrition, Physical activity, Cognitive behavioral, Hispanic

## Abstract

**Background:**

Hispanic women and children who become overweight or obese are at risk for developing prediabetes, type 2 diabetes, and cardiovascular disease later in life. Interdisciplinary interventions which target Hispanic women and their 3–5-year old children to improve nutrition and physical activity behaviors, manage adiposity and weight in mothers, and prevent excessive adiposity and weight gain trajectory in their children offer promise to break the intergenerational cycle.

**Methods:**

Using a randomized two-group, repeated measures experimental design, the goal of the proposed study is to investigate the efficacy of a 12-week nutrition and physical activity program including education, coping skills training, and home-based intervention in Hispanic women and their 3–5-year old children. The program includes 6 months of continued monthly contact to help overweight and obese Hispanic mothers and their children improve adiposity, weight (trajectory for children), health behaviors (nutrition and physical activity), and self-efficacy We will partner with two federally qualified health departments in Durham and Chatham counties, North Carolina to enroll participants. We will partner with community centers to deliver the intervention. A total of 294 Hispanic women with a BMI ≥ 25 kg/m^2^ and 294 Hispanic 3–5-year old children with a ≥ 25th BMI percentile will be enrolled over 4 years and randomized to the experimental or equal attention control group. Data will be collected at Time 1 (0 months [baseline]) to Time 2 (9 months [completion of the intervention]) and Time 1 to Time 3 (15 months [after 6 months with no contact from the study staff]). Data collected will include adiposity and weight in mothers and children (primary outcomes). Secondary outcomes will include health behaviors and self-efficacy in the mothers and in the children. We will also evaluate the cost of delivering the program for public health departments. We will use general linear mixed models to test the hypotheses.

**Discussion:**

Decreasing overweight and obesity in Hispanic women and slowing adiposity and weight gain trajectory in young Hispanic children is urgently needed to decrease morbidity, mortality, and future health care costs.

**Trial registration:**

NCT03866902. (March 7, 2019).

## Background

There is an urgent need to prevent excessive adiposity and weight gain trajectory in Hispanic children. In the United States (U.S.), overweight (BMI ≥ 85th and < 95th percentile) and obesity (≥ 95th percentile) are prevalent in both genders, affect all ages, and cross all ethnic groups, though minority children from families with lower incomes and less education are particularly high-risk [1]. The 2011–2012 prevalence of overweight and obesity in 2-to-19 year old Hispanic youth is 38.9%, non-Hispanic Black youth is 35.2%, while non-Hispanic White youth is 28.5% [1]. Obesity increases risk for developing prediabetes, type 2 diabetes, and cardiovascular disease [2, 3]. Childhood obesity is estimated to cost $14 billion annually in direct medical costs in the U.S. [4, 5].

There are currently 55.3 million Hispanics living in the U.S. [6]. In 2016, Hispanics accounted for 9% of the population in North Carolina [6]. Overweight and obesity in Hispanic mothers and their children involve a complex interplay between acculturation, the food environment, the built environment, the influence of media, and a lack of support [7]. Many Hispanic men work long hours, while mothers stay home to care for their children without close family support [7]. Greater acculturation is significantly associated with increased consumption of fast foods and decreased moderate-to-high intensity exercise [8, 9]. Many communities where Hispanics live lack access to healthy affordable food and have only one-third as many supermarkets as other communities [10]. Confronted with an abundance of low cost, calorie-dense, high fat food, and limited access to fresh produce, Hispanics increase their intake of calories and fat and decrease their intake of fruits and vegetables [7, 11].

Transportation, infrastructure, and safety issues limit Hispanic mothers’ and children’s options for physical activity. Mothers engage in less physical activity than in their home country where most did not own cars, televisions, or computers and where they were more likely to include physical activity in their everyday life [7, 11]. Hispanic parents also report barriers to their children’s physical activity [12], including neighborhood safety and transportation problems [12]. Hispanic children are less likely than non-Hispanic White children to be involved in organized physical activity outside of school [12]. The media also contributes to the lack of healthy eating and physical activity. In 2015, advertisers spent more than $7.8 billion on restaurant, food, and beverage advertising targeting Hispanic households [13]. Hispanic children spend more time watching television than non-Hispanic White children (321 min per day versus 216 min) [14], and the number of hours spent watching television is positively associated with overweight and obesity [15, 16]. We will work with mothers to help them increase their own and their children’s physical activity.

Mothers influence children’s nutrition and physical activity behaviors by modeling health behaviors, structuring children’s activities, and selecting content and portion sizes of meals [17–20]. Mothers have more influence when children are in the preschool stage than in later stages of development [21]. Three to 5 year olds are able to pay attention to activities, think in logical steps, have good language skills, run, jump, skip, and socially and emotionally start to share, cooperate, and take turns [22]. We propose to teach mothers strategies to improve their own nutrition and physical activity behaviors, build their self-efficacy for these behaviors, and learn how to help their children eat healthier and increase physical activity. We will focus on 3–5- year-olds because our pilot work indicates that they are developmentally more prepared for intervention than younger children [23].

Nutrition and physical activity programs used in the general population that were found to be most effective incorporated both behavioral and cognitive strategies and included parental involvement [19, 24, 25]. To date, we have found no interventions that have been developed to help Hispanic mothers who are overweight or obese manage their weight and prevent excessive adiposity and weight gain in their young children using a culturally and language tailored intervention*.* We propose to test the efficacy of nutrition and physical activity education, coping skills training, and physical activity intervention (Healthy Mothers-Healthy Children Intervention) tailored to Hispanic mothers and using an age-appropriate nutrition and physical activity intervention for their children (Color Me Healthy) [26]. Coping skills training includes social problem solving, cognitive restructuring, assertiveness training and conflict resolution to improve self-efficacy [27–31]. We will provide support as mothers increase their self-efficacy, practice new nutrition and physical activity behaviors, and help their children eat healthier and increase physical activity.

## Methods/design

The aims of the study are as follows.

Aim 1: Test the efficacy of the intervention in slowing adiposity and weight gain trajectory and improving health behaviors (nutrition and physical activity) in 3–5-year-old Hispanic children from Time 1 (0 months [baseline]) to Time 2 (9 months [completion of the intervention]) and Time 1 to Time 3 (15 months [after 6 months with no contact from the study staff]).

Aim 2: Test the efficacy of the intervention in decreasing adiposity and weight and improving health behaviors and self-efficacy in overweight or obese Hispanic mothers from Time 1 to Time 2 and from Time 1 to Time 3.

Primary outcomes include slowing of child adiposity (waist circumference and triceps and subscapular skinfolds) and weight gain (BMI percentile) trajectory and a decrease in mother’s adiposity and weight (BMI). We hypothesize that intervention group children will slow the trajectory of adiposity and weight gain, and intervention group mothers will decrease adiposity and weight significantly more than the control group. Secondary outcomes include child and mothers health behaviors (nutrition and physical activity) and mother’s self-efficacy. We hypothesize that intervention group children and mothers will improve health behaviors and mothers will improve self-efficacy significantly more than the control group.

Aim 3: Identify the mediators through which the intervention influences the trajectory of adiposity and weight gain in children and decrease in adiposity and weight in mothers. We hypothesize that health behaviors and self-efficacy may mediate adiposity and weight loss in mothers and nutrition and physical activity behaviors in children may mediate a slower adiposity and weight gain trajectory.

Aim 4: Calculate the cost of implementing the intervention for public health departments and the costs for mothers participating, from Time 1 to Time 2 and compare these costs to the outcomes of the intervention (cost-effectiveness) and the potential economic benefits of these outcomes (cost-benefit analysis). We hypothesize that the intervention is primarily focused on knowledge and behaviors in an at-risk population and may be cost-effective in comparison with other approaches to managing overweight and obesity.

Exploratory Aim: Determine whether intervention group mothers in the overweight versus obese BMI category benefit equally from the intervention in adiposity, weight, health behaviors, and self-efficacy.

This study will use a two-group, randomized repeated measures study design to evaluate the efficacy of the intervention with 294 dyads of Hispanic mothers with overweight and obesity and their 3–5-year-old children. The mothers in the Healthy Mothers-Healthy Children intervention group will receive a 12-week intensive intervention and 6 months of continued support. The intensive intervention will include 60 min of nutrition and exercise education and coping skills training, and a 45 min physical activity class and a home-based physical activity program handout each week for 12 weeks. The continued support classes for the mothers in the intervention group will include 60 min of classroom discussion regarding problems they are having with nutrition and physical activity for themselves and their children, and a 45-min physical activity class monthly for 6 months following the intensive intervention. The children in the intervention group will receive an intensive intervention, which will include 60 min of Color Me Healthy classes [26] and 45 min of free play weekly for 12 weeks. The Color Me Healthy curriculum is focused on fun interactive nutrition and physical activity which are designed to engage young children using color, music and exploring their environment [26]. The continued support classes for the intervention children will include a review of the Color Me Healthy classes [26] and 45 min of free play monthly for 6 months following the intensive intervention. Each weekly or monthly class for mothers and children is a total of 105 min. The equal attention control group mothers will receive English as a Second Language (ESL) classes for 105 min for 12 weekly classes and then monthly classes for 6 more months. The equal attention control group mothers will receive homework assignments to help them practice their English language skills. The homework handouts will be at the same frequency as the Healthy Mothers-Healthy Children intervention group mothers. The equal attention control group children will receive 105 min of coloring with crayons and being read stories for 12 weekly classes and then 6 monthly free play classes.

Data will be collected at Time 1 (0 months [baseline]), Time 2 (9 months [completion of the intervention]), and Time 3 (15 months [after 6 months with no contact from the study staff]). Time 2 data will determine the magnitude of the intervention effects after the intensive intervention and continued support; Time 3 data collection will test efficacy after the mother-child dyads have had sufficient time to implement their new nutrition, physical activity, and coping skills on their own. These times were chosen because 3 to 6 months after completion of an intervention represents a standard length of time for follow-up in weight reduction studies, and gives mothers and children the time necessary to make changes in health behaviors [19].

### Setting

The study will build upon established partnerships between The University of North Carolina at Chapel Hill, the Chatham County Health Department, and the Durham County Health Department. Bilingual research assistants (RAs) will distribute brochures, give mothers information about the study, answer questions, and enroll mother-child dyads. This approach worked well in previous studies [32, 33]. Specifically, over 4 years we will enroll 36–38 dyads for each cohort in each site in each of eight rolling enrollment periods that each last 1 month.

We will rent space for the intervention from community centers with classrooms and playgrounds in Chatham and Durham Counties. The public health departments and community centers are situated in communities where the participants live and are either within walking distance from their homes or on bus lines, increasing the probability of successful recruitment and retention for the proposed study. In previous studies, the mothers drove to the community centers together [23].

### Participants

Inclusion criteria for mothers will be age 18 years or older; self-identification as Hispanic; a BMI ≥25 kg/m^2^; residence with the child; ability to understand spoken Spanish; and able to consent to join the study and consent for their child to join the study. Inclusion criteria for children will be age 3–5-years; ability to understand spoken Spanish; and ≥ 25th BMI percentile for age and gender. Limited English proficiency (LEP) will be assessed and low acculturation will be measured using the Short Acculturation Scale for Hispanics [34] with a score from 1.00 to 2.99; Mothers will fill out a health history questionnaire to ascertain if they have a heart murmur, congenital heart disease, family history of sudden death, difficulty exercising or psychological problems that would prevent participation. A sports physical will be conducted by a bilingual nurse practitioner on all mothers during baseline data collection to ensure that they have no medical problems requiring physical activity restrictions. If a mother answers yes to any of these health history questions or the nurse practitioner finds a heart murmur or any other concerns, she will be excluded from the study and referred to a health care provider.

### Sample size calculation

A total of 250 mother-child dyads, or 125 dyads in each randomized group, are expected to complete the study. However, we will enroll 294 mother-child dyads (*n* = 588 participants) or 147 dyads per group, to take into account a 15% attrition rate. Our previous studies with Hispanic mothers with LEP and their children have had attrition rates ranging from 9 to 15% [23, 33]. Power calculations were performed with POWERLIB20 SAS/IML modules, which incorporate methods described in Muller [35]. These methods calculate power for a general linear multivariate model that includes repeated measures data structures, of which a two-group longitudinal design is a special case. Power was calculated on the use of a separate multivariate model for each outcome addressed in the aims, incorporating measurements at all available time points. Effect size estimates were based on the pilot data [23], where estimated within-subject correlation and standardized mean difference were .25 and .53, respectively, for mothers’ BMI and .30 and .59, respectively, for children’s BMI percentiles. For each outcome, power was computed for a test of the time by treatment interaction at a two-sided significance level of .05. For an analyzable sample of 125 mother-child dyads per group (total of 250 mother-child dyads), calculated statistical power exceeds 95% for both the test of children’s BMI percentiles in Aim 1 and the test of mothers’ BMI in Aim 2; if the within subject correlations and standardized mean differences are lower than observed in the pilot at .20 and .45, respectively, statistical power will nonetheless exceed 80%. A formal power analysis was not conducted for Aim 3 because the focus is on estimation (rather than testing) of direct and indirect mediation effects. Nonetheless, based on Shrout and Bolger [36], a sample size of 80 to 85 should provide power in the 70 to 85% range to detect medium effects of the intervention on the mediator (e.g., maternal self-efficacy), and the mediator on the outcomes (e.g., mother’s BMI) with a two sided .05 significance level. Our 250 dyads should provide an adequate sample to quantify mediation of the relevant variables.

### Procedures for minority recruitment and enrollment and challenges

We will hire only bilingual (Spanish/English speaking) staff, and all recruitment and intervention materials and instruments will be in Spanish. Two months before enrollment, the bilingual project manager will give a presentation to staff at both public health departments and place Spanish language brochures in each waiting room. The brochures will describe the benefits of good nutrition and physical activity and state the eligibility criteria for study participation. Bilingual RAs will be available in the waiting rooms on clinic days to share information about the study with Hispanic mothers. Face-to-face information classes worked well in our feasibility and pilot studies and we were able to enroll a sufficient number of participants each week [23]. If mothers are interested, the RAs will privately record their name and telephone number and schedule them for an enrollment screening telephone call. This technique also worked well in our pilot study, which included mother-child dyads with LEP [23]. Of 82 mother-child dyads approached in the pilot, 68% (*n* = 56) consented to participate over 1 month [23]. We plan to enroll approximately 10 mother-child dyads per week. However, we have built into the timeline additional time for enrollment for each cohort if needed. If we have any difficulty enrolling participants, we will work with both health departments to seek advice on how to improve enrollment in the study.

The bilingual project manager and bilingual staff will conduct a private telephone screening and ask mothers’ height and weight and their child’s gender, birthdate, height, and weight, calculate BMI for mothers and BMI percentile for children. If both mother and child meet the study criteria, an appointment will be made to meet at the community center to confirm eligibility; the bilingual RAs will measure height and weight and confirm BMI and BMI percentile. If eligible, the RA will explain, in Spanish, the study, requirements of participants, and the risks and benefits of participating, and random assignment; and all questions will be answered. Only the mother and one 3–5-year-old child will be eligible to join the study. Childcare will be available for other children the mother brings with her in addition to her 3–5- year-old child. Data collection with mother-child dyads will occur in the morning or early afternoon when the mother’s older children will be in school. Physiological data collection will be done in a private room. Bilingual RAs will collect the following data in the same order: health history questionnaire, sports physical, height, weight, waist circumference, triceps and subscapular skinfolds. The mothers will fill out their questionnaires in a group with a bilingual RA who will read all questionnaires in Spanish. Data collection will take 75 min for each mother-child dyad.

### Procedures for minority retention and challenges

Another challenge will be retention. Weight management studies typically have attrition rates ranging from 10 to 30% [19]. To strengthen retention, our classes will be interactive, with culturally and linguistically tailored content developed to engage and sustain the interest of the mother-child dyads. Mothers who miss class will be offered a make-up class over the phone, and reminded when the next class will be held. Additional incentives will include childcare for other children who accompany mothers and their 3–5- year-olds to class and recipes and handouts in Spanish on how to improve their children’s nutrition and physical activity behaviors. Mothers will be asked for telephone numbers of several family members and permission to call them if we cannot reach them. We will be as flexible as possible in scheduling enrollment and data collection appointments and provide tokens of appreciation such as birthday cards and a quarterly newsletter with updates on the study. Mothers in both groups will receive $50 after each data collection. These approaches proved successful in our pilot study with Hispanic mothers with LEP, where attrition was only 9–15% [23] and those mothers who stopped coming had moved out of the state. We will make every effort to keep our attrition rate lower than the projected 15%. If retention rates drop below 85%, we will contact the mothers to ask why they stopped coming and develop strategies to meet the needs of each mother to assist them to continue in the study if possible.

### Intensive intervention for mothers

The intensive intervention classes were co-created with Hispanic mothers with LEP [23, 37, 38]. Classes will be run by a bilingual interventionist and will be 60 min long with 18–19 mothers. We chose 18–19 mothers since these mothers will be enrolled together and this cohort approach worked well in our previous studies [23, 38]. Classes will include nutrition and physical activity education and coping skills training using problem solving, assertiveness training, cognitive restructuring, and conflict resolution around problems of nutrition and physical activity for mothers and their children. The nutrition classes will be based on the dietary guidelines and the Mediterranean diet will include content on understanding calories, protein, carbohydrates and fat, making healthy substitutes that are culturally acceptable, choosing healthy foods when eating out at a favorite fast food restaurant, using actual menus, and understanding how portion control can make a difference for them and their children using food models and food labels in Spanish. Mothers will receive information on how to shop as economically as possible and how to shop at local farmers’ markets, which are geographically close to the mother’s homes, where they may have better access to fresh fruits and vegetables. At the end of each class, mothers will be asked to set a nutrition or physical activity goal for the coming week for themselves and their 3-to-5-year-old child.

Physical activity classes will be held for 45 min after the intensive intervention classes and will include a warm-up and then activities such as Zumba, Yoga, walking, use of light weights and stretch bands, and a cool-down. The bilingual interventionist will reinforce the importance of physical activity and ways to increase physical activity for mothers and their children. The importance of decreasing sedentary behaviors for mothers and children will be reinforced in all classes. During the first week, each intervention mother will receive a pedometer to use as a part of the intervention. Mothers will be encouraged to increase their physical activity weekly by small increments until they are physically active 30 to 60 min a day on most days of the week with their 3–5-year old child. Missed physical activity classes will not be made up. Mothers in the intervention group will be encouraged to develop their own home-based physical activity program using suggestions given in the physical activity class and a home-based physical activity handout in Spanish given after each physical activity class to increase their and their child’s physical activity. The handouts will be reviewed with the mothers and physical activities demonstrated by the bilingual interventionist.

### Intensive intervention for children

The children’s classes will include a 60-min session plus 45 min of supervised free play with 18–19 children at the same time their mothers are in class. Each class will have 1 teacher and 3 teacher assistants to ensure safety. The Color Me Healthy [26] curriculum, which consists of 12 classes in Spanish, was purchased and used for the pilot study [23]. All of the classes are at a 3–5- year-old developmental level and include a CD with music and a song about the class of the week, colorful picture cards, and opportunities for the children to try new fruits and vegetables. Each week the bilingual interventionist will give mothers a Color Me Healthy [26] handout in Spanish that includes strategies for feeding their children healthier meals and snacks and increasing their daily physical activity. While their mothers are in physical activity class, the children will have 45 min of physical activity (supervised free play) in a playground at the community center or dance and actively play with the bilingual interventionist if it is raining.

### Continued support

During continued support, intervention mother-child dyads will return to the community center for classes once a month for 6 months. The mothers will receive a calendar at the completion of the intensive intervention classes with the dates and times and a reminder phone call several days before each class. As a part of the intervention, mothers will be weighed and then have the opportunity to engage in a discussion run by the bilingual interventionist, who will help mothers solve problems they have encountered related to nutrition and physical activity for themselves and their children for 60 min and then receive a 45 min physical activity class. The children will meet with a bilingual interventionist to review a class from the Color Me Healthy [26] curriculum for 60 min and then receive 45 min of free play. If a mother and child miss a class, the bilingual interventionist will call and ask how the mother and child are doing and give the date of the next class. Continued support classes will not be made up.

### Fidelity of the intervention

We have structured the fidelity of the intervention using the National Institute of Health (NIH), five category Treatment Fidelity Framework [39]. For category one, treatment design, both the intervention and control condition will receive different content but will be the same length, number, and duration of contact over time. The bilingual interventionists will have experience teaching health interventions, and the theoretical model is clearly articulated. For category two, training, the bilingual interventionists will be trained by the principal investigator (PI) and teach back the intervention to the PI. For category three, delivery of the intervention, integrity will be assessed by observation of two randomly selected classes per month by the project manager who will use a checklist to score classes based on pre-identified content, and drift will be defined as teaching less than 80% of protocol content. If drift occurs, the bilingual interventionists will be retrained until the protocol is followed consistently. We will also use a list of behavioral indicators to assess the interventionists’ skills in facilitating classes, engaging mother-child dyads, role playing, problem solving, providing positive feedback, and goal setting. Retraining will be provided if problems are found. In our pilot study [23], we had little difficulty in maintaining consistency of the intervention. The bilingual interventionists will collect data on attendance at each class, reasons for non-attendance, and make-up classes provided. Category four, receipt of treatment, will be evaluated during the class by the bilingual interventionist who will assess understanding and answer all questions. Category five, enactment of intervention skills, will be evaluated during the six monthly continued support classes by the bilingual interventionist.

### Equal attention control group

Mothers in the control group will receive English as a Second Language (ESL) classes taught by professional ESL teachers; they will receive the same number of contacts and time as the intervention group mothers (105 min weekly for 12 weeks and 105 min monthly for 6 months). The ESL classes will be offered because when exit interviews were conducted after the pilot study [23], mothers were asked if they “were not in the intervention group, what type of classes would they want?” The mothers (90%) wanted ESL classes to improve their English. Providing these classes to the control group most likely will improve enrollment and retention. Children in the control group will color with crayons and have stories read and will receive the same number of contacts and times as the children in the intervention group (105 min weekly for 12 weeks and 105 min monthly for 6 months). The control classes will be held on separate days of the week from the intervention classes in the same community centers. Data will be collected from the mother-child dyads in the control group at Time 1, Time 2, and Time 3; and the mothers will receive $50 each time. Mothers will be called several days before classes and data collection to remind them. Mothers will receive a pedometer after they have completed Time 3 data. Attrition rates in our previous studies were equal in the intervention and control groups and ranged from 9 to 15% [23].

### Measures

All instruments are in Spanish and have been adapted for mothers with LEP and used and evaluated in our feasibility and pilot studies [23] and found to have excellent reliability and validity. Bilingual RAs blinded to study group assignment will collect data. The PI will train the project manager, who will train the RAs using a standardized system for collecting physiologic and questionnaire data. We will develop a training manual for collecting physiological data and administering questionnaires, and copies will be given to the RAs. To ensure inter-rater reliability, RAs will be trained and tested for inter-rater reliability prior to each data collection on height, weight, waist circumference, and triceps and subscapular skinfolds. Questionnaires will be read to the mothers by a bilingual RA. As a part of the Time 1 data collection, mothers will be asked to fill out a Health History Questionnaire and will receive a physical to make sure they do not have any medical conditions that would exclude them from joining the study.

#### Weight status

In a private room, height and weight will be measured twice on all children and mothers in street clothes without shoes and then averaged. Height will be measured using a stadiometer, calibrated in 1/8-cm (cm) intervals. Weight will be measured to the nearest 0.1 kg using a Tanita WB-110A Digital Scale. For children, BMI percentiles will be calculated via standard computer software by inputting height, weight, age, and gender [40, 41]. Children with a BMI ≥ 85th and < 95th percentile for age and gender are overweight, and those at or above the 95th percentile are classified as obese [43]. BMI in mothers will be calculated via standard computer software, by entering height and weight [41]. Overweight is defined as a BMI 25.0 kg/m^2^ to 29.9 kg/m^2^ and obesity is defined as a BMI ≥ 30.0 kg/m^2^ [42].

#### Adiposity

Waist circumference in children and mothers will be measured as in “The Insulin Resistance Atherosclerosis Study (IRAS)” using a Figure Finder measuring tape (Novel Products Inc., Rockton, IL) [43]. Two RAs will measure waist circumference in mother-child dyads three times and averaged following the National Health and Nutrition Examination Survey procedures [44–47]. Skinfolds will be measured in mother-child dyads on the right side of the body using Lange skinfold calipers. Measures will be taken three times and averaged. Two RAs will do triceps and subscapular skinfolds, with one RA measuring and one recording, as recommended by National Center for Health Statistics [48]. Triceps and subscapular skinfold thickness will be calculated using the gender-and race-specific methods of Slaughter et al. [49].

#### Health behaviors

The Adult Health Behavior Survey [50] was developed to examine trends in nutrition intake over time. Questions include: How many times do you eat breakfast per week? How many times to you eat fast food each week? How many servings of fruits and vegetables do you eat each day? All instruments have been tested in the pilot study [23] and found acceptable for Hispanic mothers. The Health Promoting Lifestyle Profile II (HPLP II) will be used to measure health promoting lifestyle behaviors in mothers [51]. This 48-item, 4-point Likert scale questionnaire contains statements about personal habits, with 4 response choices: never, sometimes, often, or routinely. We will use the scores for the health responsibility, physical activity, nutrition, and stress management subscales. The 24-Hour Dietary Telephone Food Recall is the “gold standard” for collection of individual dietary data [52]. A bilingual RA will complete a 24-h dietary food recall for all mothers and for their children by phone within 1 week after Time 1-Time 3 data collection; data will be collected for 2 days during the week and 1 weekend day since many individuals eat differently during the week and on the weekend [52]. The RA will follow the procedures established in the pilot study, and enter the data into NutritionistPro [53], which allows entry of multi-day diet recall organized by day and/or meal, diet recall averaging, and a variety of reports.

Accelerometry will be measured for 7 days in mothers and children at Time 1-Time 3 using the ActiGraph GT3X+ accelerometer worn on the wrist to capture physical activity. Accelerometers can estimate the intensity, frequency, and duration of physical activity [54, 55]. Among adults and 3–5- year old children, 5–7 days of monitoring are required to reliably estimate physical activity [55–58]. The bilingual RA will explain the waterproof watch to mothers, asking them to wear it for 24 h/day for 1 week. Mothers will be provided with a phone number to call if they have questions. There will be a drop box at each community center where the mothers will drop off the accelerometers after 7 days.

#### Self-efficacy

The Eating Self-Efficacy Scale [59] will be used to measure nutrition self-efficacy in mothers. This 25-item instrument asks respondents to rate their difficulty in controlling eating from 1 (no difficulty) to 6 (very difficult) on two subscales, negative affect and socially acceptable circumstances. Negative affect eating is related to emotional eating and socially acceptable eating is related to overeating at family events and holidays. Answers are reverse scored to reflect 1 as very difficult and 6 as no difficulty. The Exercise Self-Efficacy Scale [30] will be used to record the strength of efficacy beliefs using 18 questions on a 10-point scale, ranging in 1-unit intervals from 0 (cannot do at all) through intermediate degrees of assurance such as 5 (moderately certain can do) to 10 (certain can do) [30]. Scores are summed and then divided by 18 to calculate a total score. Higher scores indicate greater exercise self-efficacy.

#### Sociodemographic questionnaire

Mothers will complete a demographic data sheet for themselves and their children. Information on mothers will include age, marital status, employment status, socioeconomic status and education level. Questions for mothers to answer about their children will include age, birth order, and their general health. Mothers will fill out a Health History Update Questionnaire. A questionnaire at Times 2 and 3 to ask if they or their children have developed asthma, diabetes, started steroids, or started psychiatric medications.

#### Process evaluation guide

The project manager will observe two randomly selected classes per month using guide to assess whether classes are engaging the mother-child dyads and being taught according to the protocol.

#### Exit interview

At Time 3 data collection, an interview will be conducted with each intervention group mother to elicit information about what mothers liked or disliked about the intervention.

### Data management

Participants will be tracked using ID numbers. All data will be double entered into REDCap by different RAs with built-in range checks and skip patterns. Comparisons will be run on the two versions, and inconsistencies will be checked against the raw data and corrected. Data will be verified and stored in a secure server. Data will undergo range, consistency, and outlier checks. All data decisions will be recorded in a logbook with an audit trail. SAS datasets will be created for analysis.

### Data analysis

Means, standard deviations, minimums, medians, and maximums will be determined for each continuous variable; frequencies and percentages will be tabulated for each categorical variable. Preliminary analyses will be performed to determine whether, despite randomization, the intervention and control groups were unbalanced on gender of the age of the child, age of the mother and child, or family income. Any variable with an imbalance between the groups at baseline will be examined to determine whether it is related to any of the outcome variables. If significant relationships are identified, the variable will be included as a covariate in the models for the affected outcome(s) as a potential confounder. An intent-to-treat analysis will be used in which all subjects are included in the analysis and analyzed according to their initial randomized assignment.

Aim 1: To test the efficacy of the intervention in decreasing trajectory of adiposity and weight gain in 3-5 year-old children, separate mixed models will be used to test longitudinal differences between the intervention and control group means for each outcome across the study period. Mixed effects models, with a random intercept to account for between-mother or between-child differences, will be used for repeated measures analyses of each outcome (child BMI percentile, waist circumference, and triceps and subscapular skinfolds). The dependent variable will be will be the outcome at each of the three time points; the model will include time point (categorical), intervention group, and the interaction between the two. A test of this interaction will serve as the overall test of intervention effect, and step-down tests will be conducted to compare Time 2 and Time 3 to Time 1, and to estimate how or whether the intervention effect changed from Time 2 to Time 3, using a Hochberg correction to account for multiple comparisons [60, 61]. Results will be summarized through the least-squares means for each group at each time point and the mean difference and corresponding 95% confidence interval.

Aim 2: To test the efficacy of the intervention in decreasing adiposity and weight in mothers, we will fit mixed models analogous to those described for Aim 1 with each of the primary outcomes (mother’s BMI and adiposity measures: waist circumference, and triceps and subscapular skinfolds) and secondary outcomes (child health behaviors: nutrition and physical activity, mother’s health behaviors: nutrition and physical activity, and mother’s self-efficacy: nutrition and physical activity). Aim 3: To identify the mediators through which the intervention influences weight management in mother-child dyads, the approach of Shrout and Bolger [36] will be employed. This method, based on bootstrapping, builds on established mediation testing procedures, such as the Baron and Kenny approach [62] and the Sobel test [63]. Mediation analyses test pathways by which an explanatory variable (X) is theorized to influence the mediator (M), which in turn influences the outcome (Y). Models will be conceptualized for each measure of weight and adiposity for mothers identified in Aim 1 (Y) and for each proposed mediator (M), relative to the randomized intervention group (X). For each model, an effect ratio (defined as the ratio of the indirect effect to the total effect, yielding the proportion of the effect that is mediated) and its bootstrapped distribution will be computed to assess the strength of mediation. Mediators to be tested are improvements in health behaviors and self-efficacy. For example, we will analyze the effect of the intervention on change in maternal self-efficacy from baseline to Time 2, and, in turn the effects of this change in maternal self-efficacy at Time 2 on change in maternal BMI at Time 3.

Sensitivity analyses for Aims 1–3. Site will be included in all the models, and we will explore for evidence of differential effects across cohort or site. The primary analyses use all available data, but if there is evidence of differential missingness by treatment group, we will use imputation methods as a sensitivity analysis.

Aim 4: We will calculate the incremental cost-effectiveness of the intervention, carried out from society’s perspective, as:

$$ C{E}_{ij}=\frac{C_i-{C}_j}{E_i-{E}_j} $$

Where C is cost over the time period of the study; E is effectiveness measured by the outcomes; *i* refers to the intervention and *j* refers to the control group. We will calculate cost-effectiveness for the principal outcomes of the study; including cost per unit of BMI reduction, and cost per unit of improvement on the nutrition and physical activity self-efficacy scales. We will carefully track and calculate the costs of the program intervention activities and the control group activities, in order to measure the differences between the costs in the two groups. These costs will include hiring interventionists for all aspects of the Healthy Mothers-Healthy Children intervention. We will track the time invested in the program by participants in both the intervention and control group and calculate the value of time (opportunity cost) relative to the mothers’ average income. Exploratory Aim: Analyses will be performed on weight status, adiposity, health behavior, and self-efficacy to explore whether the intervention effects for mothers are similar for those who are overweight (BMI 25–29.9 kg/m^2^) and those who are obese (BMI ≥30 kg/m^2^). The mixed models described for Aims 1 and 2 will be implemented for each of the weight status, adiposity, health behavior, and self-efficacy variables, with the addition of four explanatory variables to each model: an indicator for the obese group, the interaction between this indicator and the indicator variable for group membership, the interaction between this indicator and time, and the three-way interaction among the group indicator, obese indicator, and time. The hypothesis test for the three-way interaction as well as the obesity indicator by group indicator interaction jointly equal to zero will indicate whether the intervention effects are homogeneous across BMI ranges. For each outcome, differences will be calculated to test the effects of the intervention versus control group at each follow-up point separately for each BMI range. Similar analyses will be performed with groupings of children in the 25th–84.9th percentile (normal weight), 85th–94.9th percentile (overweight) and ≥ 95th percentile (obese).

## Conclusion

The Healthy Mothers-Healthy Children study will provide data on the evidence of management of overweight in Hispanic mothers and prevention of excessive weight gain in 3-to-5-year old Hispanic children. This study has the potential to change management of mothers and children and provide public health departments with a valuable program to improve outcomes in Hispanic mothers and children.

## Data Availability

Data sharing is not applicable to this manuscript since no datasets have been generated or analyzed to date. Data will be available after completion of the study upon reasonable request to the Principal Investigator, Dr. Diane Berry. The aggregate data will be deposited in the National Institutes of Health and National Institute of Nursing Research data repository.

## Abbreviations

BMI: Body mass index
ESL: English for Second Language
LEP: Limited English Proficiency
NIH: National Institutes of Health
PI: Principal Investigator
RA: Research assistant
